# *REST*, a master regulator of neurogenesis, evolved under strong positive selection in humans and in non human primates

**DOI:** 10.1038/s41598-017-10245-w

**Published:** 2017-08-25

**Authors:** Alessandra Mozzi, Franca Rosa Guerini, Diego Forni, Andrea Saul Costa, Raffaello Nemni, Francesca Baglio, Monia Cabinio, Stefania Riva, Chiara Pontremoli, Mario Clerici, Manuela Sironi, Rachele Cagliani

**Affiliations:** 1 Bioinformatics, Scientific Institute IRCCS E. MEDEA, 23842 Bosisio Parini, Italy; 2 Don C. Gnocchi Foundation ONLUS, IRCCS, 20148 Milan, Italy; 3 Department of Physiopathology and Transplantation, University of Milan, 20090 Milan, Italy

## Abstract

The transcriptional repressor REST regulates many neuronal genes by binding RE1 motifs. About one third of human RE1s are recently evolved and specific to primates. As changes in the activity of a transcription factor reverberate on its downstream targets, we assessed whether REST displays fast evolutionary rates in primates. We show that REST was targeted by very strong positive selection during primate evolution. Positive selection was also evident in the human lineage, with six selected sites located in a region that surrounds a VNTR in exon 4. Analysis of expression data indicated that *REST* brain expression peaks during aging in humans but not in other primates. Because a *REST* coding variant (rs3796529) was previously associated with protection from hippocampal atrophy in elderly subjects with mild cognitive impairment (MCI), we analyzed a cohort of Alzheimer disease (AD) *continuum* patients. Genotyping of two coding variants (rs3796529 and rs2227902) located in the region surrounding the VNTR indicated a role for rs2227902 in modulation of hippocampal volume loss, indirectly confirming a role for REST in neuroprotection. Experimental studies will be instrumental to determine the functional effect of positively selected sites in *REST* and the role of *REST* variants in neuropreservation/neurodegeneration.

## Introduction

The Repressor Element 1 Silencing Transcription factor (REST, also known as neuron restrictive silencer factor, NRSF) is a transcriptional regulator that binds a specific 21 bp motif (Repressor Element 1–RE1) in the regulatory regions of target genes^1, 2^. To regulate expression, REST interacts with chromatin modifiers (HDAC complex) by recruiting corepressor mSin3 and CoREST^3, 4^.

REST acts as a negative regulator of neuronal gene expression during both embryogenesis and adult neurogenesis^5, 6^, and also plays a role in modulating synaptic plasticity^7^.

In the normal aging brain, REST is the most activated transcription factor and functions as a neuroprotective modulator^8^. In fact, REST levels increase with age in the prefrontal cortex (PFC) and hippocampus of healthy adults. REST expression correlates with the up-regulation of protective stress response genes, as well as with the repression of genes that promote cell death. These findings support the notion that REST is necessary during aging to maintain neuronal viability and to preserve cognitive functions^8^.

Moreover, REST and its target genes have been implicated in the pathogenesis of a number of different neurodegenerative diseases, including Alzheimer’s Disease (AD) clinical *continuum*, frontotemporal dementia, and dementia with Lewy bodies^8, 9^. In these pathologies, REST is depleted in the nucleus of PFC and hippocampal neurons and colocalizes in autophagosomes together with pathological misfolded proteins (e.g. Aβ, phosphorilated Tau, TDP-43, α-synuclein).

In elderly subjects with mild cognitive impairment (MCI), a disorder that has been associated with risk for dementia, a missense *REST* variant (rs3796529) was associated with baseline hippocampal volume and with the rate of hippocampal grey matter (GM) density loss, suggesting that the minor allele of rs3796529 confers a protective effect on hyppocampal morphology^10^. This finding was confirmed in a larger MCI and AD cohort; in particular the minor allele of rs3796529 was associated with larger hippocampal CA1 volumes^11^.

A polymorphic variable number tandem repeat (VNTR) in the coding region of *REST* was described and characterized as having two major alleles of 4 or 5 48-nucleotide repeats^12^. A haplotype containing the VNTR was associated with general cognitive skills in elderly subjects of European ancestry. In particular, individuals carrying the 4 repeat allele displayed higher cognitive abilities^12^.

*REST* is conserved in vertebrates and a genome-wide search for RE1 elements indicated that, during vertebrate evolution, novel RE1s have arisen and generated species-specific REST targets^13^. In particular, about one third of human RE1 elements are specific to primates. Although ancient RE1 motifs recruit REST with higher affinity, the recently evolved primate-specific RE1s have preserved the ability to recruit REST *in vivo* ^13^. In line with these data, a comparison of human and mouse embryonic stem cells identified several human-specific REST targets. These genes are enriched for memory and learning functions^14^.

These observations, together with the central role played by REST in neurodevelopment and neuropreservation, led to the suggestion that the expansion of REST targets contributed to the development of primate-specif traits in terms of brain function or cognition^13^.

Previous analyses of FOXP2, another transcription factor that is highly expressed in the brain, indicated that positive selection acted in concert on the *FOXP2* gene and on its target sequences^15^. This is conceivable as changes in the activity or expression of a transcription factor reverberate on its downstream targets. We thus analyzed the evolutionary history of *REST* in primates. Results herein show that *REST* was targeted by very strong positive selection during primate evolution, and most positively selected sites cluster in the region surrounding the VNTR. This also holds true for sites that were specifically selected in the human lineage. We also report that a *REST* haplotype comprising a variant in linkage disequilibrium with the VNTR modulates right hippocampal volume in an Italian cohort of AD subjects.

## Results

### REST evolution in primates

To analyze the evolutionary history of *REST* in primates, we obtained and aligned coding sequence information for 21 primate species available in public databases (Supplementary Table S1). Because of misalignment in the region corresponding to the human polymorphic VNTR (exon 4), this 240 bp region was filtered.

We calculated the average nonsynonymous substitution/synonymous substitution rate ratio (dN/dS, also referred to as ω) using the single-likelihood ancestor counting (SLAC) method^16^: dN/dS for *REST* amounted to 0.41 (95% confidence intervals: 0.38–0.45). Although this result indicates purifying selection (i.e. dN/dS < 1) as the major driving force in shaping *REST* gene diversity, the dN/dS value is higher than those observed for most mammalian genes^17^.

We thus tested whether positive selection (i.e. dN/dS > 1) acted on a subset of *REST* codons by applying likelihood ratio tests (LRT) implemented in the *codeml* program^18, 19^. Specifically, we used LRTs that compare models of gene evolution allowing (NSsite models M2a and M8, positive selection models) or disallowing (NSsite models M1a and M7, null models) a class of codons to evolve with dN/dS > 1.

**Figure 1:**
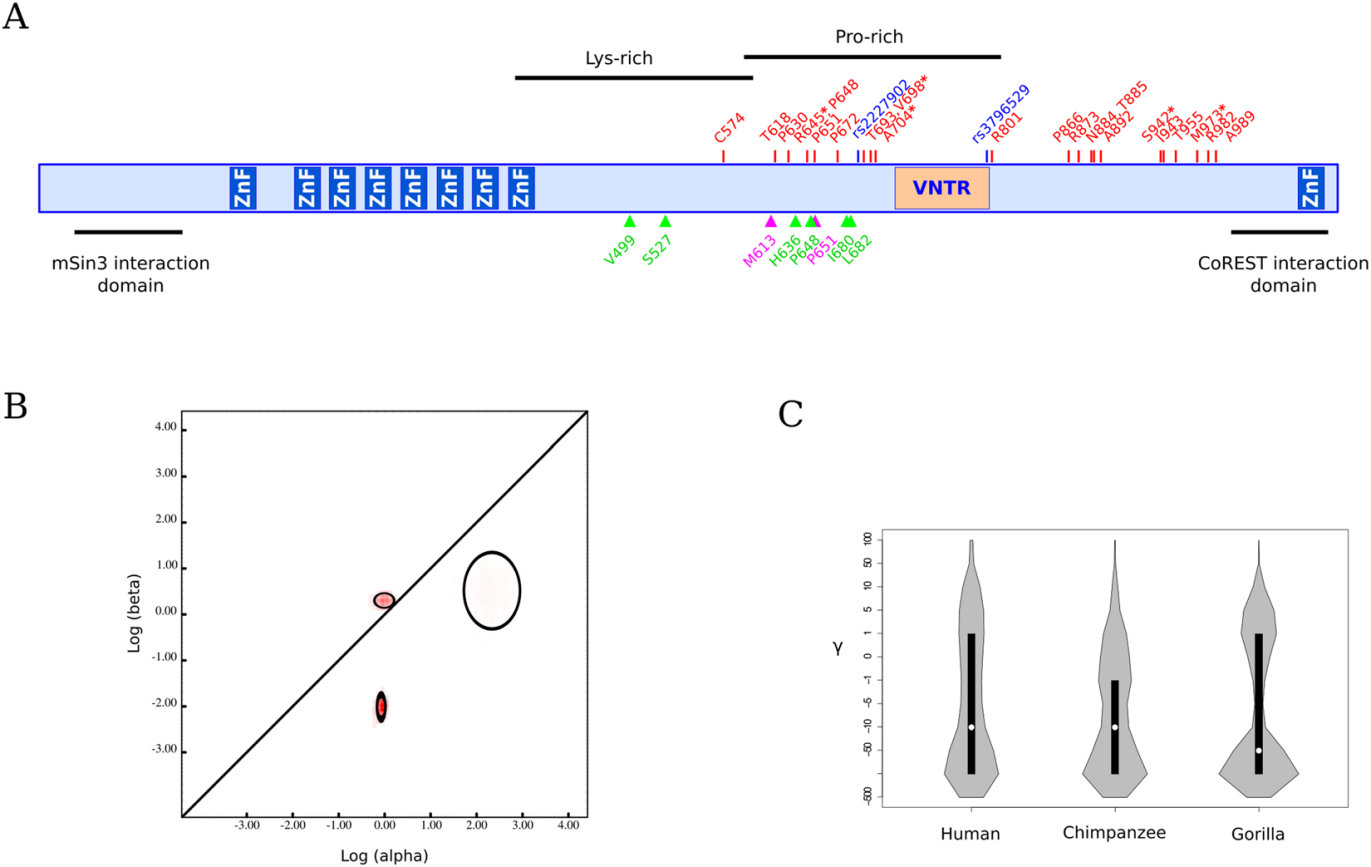
*REST* evolutionary analysis. (**A**) Schematic representation of REST protein structure. Zinc-finger domains (ZnF), a variable number tandem repeat (VNTR), protein repressor domains that recruit corepressor mSin3 and CoREST, and Lysine and Proline rich domains (Lys-rich; Pro-rich) are indicated on the structure. Positively selected sites in the primate phylogeny, in human and gorilla lineages are reported in red, green, and magenta, respectively. Asterisks denote positively selected sites identified by 3 different methods. Missense variants genotyped in AD *continuum* subjects are shown in blue. (**B**) Evolutionary fingerprinting of the primate *REST* genes. The estimate of the distribution of synonymous (α) and nonsynonymous (β) substitution rates is plotted on a log-log scale. The ellipses reflect a Gaussian-approximated variance in each individual rate estimate, and colored pixels show the density of the posterior sample of the distribution for a given rate. The diagonal line represents the neutral expectation (dN/dS = 1), points above the line correspond to positive selection (dN/dS > 1), and points below the line to purifying selection (dN/dS < 1). (**C**) Violin plots of selection coefficients (median, white dot; interquartile range, black bar) for the *REST* gene in *Homininae*. Selection coefficients (γ) are classified as strongly beneficial (100, 50), moderately beneficial (10, 5), weakly beneficial (1), neutral (0), weakly deleterious (−1), moderately deleterious (−5, −10), strongly deleterious (−50, −100), and inviable (−500).

Both null models were rejected in favor of the positive selection models; the same result was obtained using different codon frequency models (F3X4 and F61) (Table 1). In order to identify specific sites subject to positive selection, we applied the BEB, FUBAR, and REL analyses (see Methods). To limit false positives, only sites detected using at least two methods were considered as positive selection targets. A total of 22 positively selected sites were identified, 5 of which were detected by all three methods (Fig. 1A). All selected sites were located in a relatively large region surrounding the VNTR.

**Table 1:** Likelihood ratio test statistics for models of variable selective pressure among sites (F3X4 and F61 models of codon frequency).

Model	−2ΔLnL	*p value*	% of sites (average dN/dS)
**F3**X**4**			
M1a vs M2a	14.30	7.85 × 10^−4^	7.54 (2.01)
M7 vs M8	23.34	8.54 × 10^−6^	16.61 (1.80)
**F61**			
M1a vs M2a	9.24	9.83 × 10^−3^	7.54 (2.01)
M7 vs M8	16.26	2.94 × 10^−4^	22.51 (1.51)

Note: M1a is a nearly neutral model that assumes one ω class between 0 and 1, and one class with ω = 1; M2a (positive selection model) is the same as M1a plus an extra class of ω > 1. M7 (null model) assumes that 0 < ω < 1 is beta distributed among sites in 10 classes; M8 (selection model) has an extra class with ω >  = 1; 2ΔLnL: twice the difference of the natural logs of the maximum likelihood of the models being compared; *p value*: *p value* of rejecting the neutral models (M1a or M7) in favor of the positive selection model (M2a or M8); % of sites (average dN/dS): estimated percentage of sites evolving under positive selection by M8 (dN/dS for these codons).

In order to evaluate the presence of episodic positive selection acting on a subset of primate branches, we applied the BUSTED test (branch-site unrestricted statistical test for episodic diversification)^20^. No evidence of episodic diversifying selection was detected (BUSTED *p value* = 0.188).

We next performed an evolutionary fingerprinting analysis^21^, which partitions sites into selective classes and estimates dN/dS for such classes. The best fitting model had 3 rate classes, two of them accounting for the majority of codons (Fig. 1B). Specifically, 73% of *REST* codons experienced negative selection (dN/dS = 0.13) and 26% were targeted by positive selection (dN/dS = 1.38). No sites were found to be evolving neutrally (Fig. 1B).

### REST evolution in Homininae

In order to study the evolution of the *REST* gene in *Homininae* and to gain insight into the more recent selective events in specific lineages, we applied a population genetics-phylogenetics approach (gammaMap)^22^. gammaMap leverages intra-species variation and inter-specific diversity to estimate the distribution of selection coefficients (γ) along coding regions.

In humans, the polymorphic *REST* exon 4 VNTR contains 4 or 5 repeats^12^. Analysis of genomes of *Homininae* indicated that chimpanzees have 5 repeats, whereas the gorilla *REST* gene carries only four repeat copies (see Methods).

As above, the 240 bp region corresponding to the VNTR was masked before running gammaMap.

Analysis of the overall distribution of selection coefficients indicated a similar evolutionary pattern in humans, chimpanzees, and gorillas (Fig. 1C): most codons displayed γ values below −10, indicating that purifying selection drove the evolution of a major proportion of sites in *REST*.

We next used gammaMap to identify specific codons evolving under positive selection (defined as those having a cumulative probability > 0.80 of γ > 0) in each lineage. Six sites were found to represent positive selection targets in humans (Table 2, Fig. 1A). Only two selected sites were detected in gorillas and none in the chimpanzee lineage. Six selected sites fall in a proline-rich region, the other two sites fall in the lysine-rich region (Fig. 1A).

**Table 2:** Positively selected sites in the human and gorilla lineages.

Lineage	Codon	Ancestral amino acid	Derived amino acid	Pr^a^
Human	499	Met	Val	0.8004
	527	Thr	Ser	0.7975
	636	Pro	His	0.9106
	648^b^	His	Pro	0.9202
	680	Met	Ile	0.9333
	682	Pro	Leu	0.9325
Gorilla	613	Met	Lys	0.8220
	651^b^	Pro	Thr	0.8128

^a^Posterior probability of γ > 0 as detected by gammaMap; ^b^ positively selected site in both primate phylogeny and specific lineage.

### REST brain expression in primates

**Figure 2:**
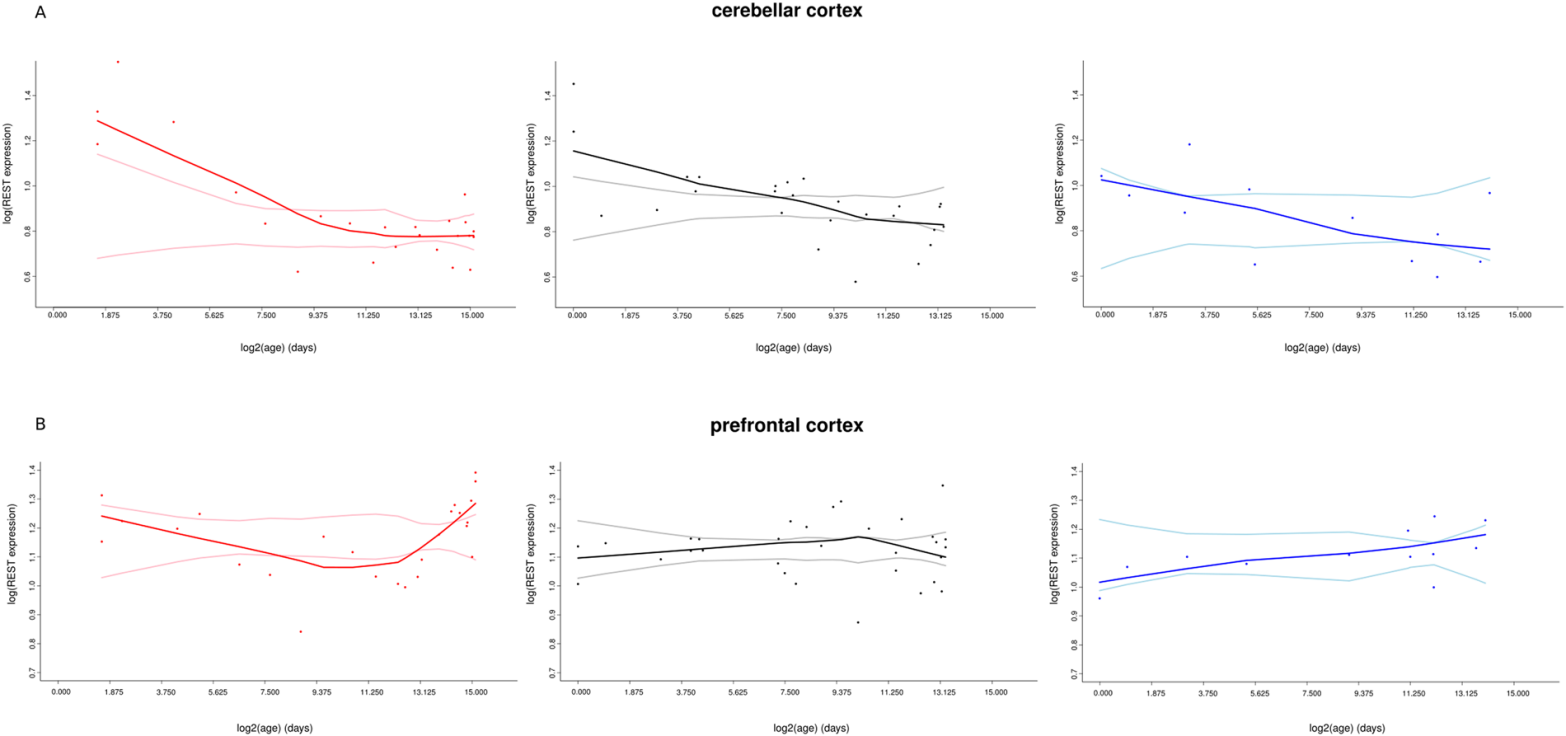
Expression profile of *REST* in the prefrontal cortex (superior frontal gyrus) and cerebellar cortex at different age points. Data are derived by Somel *et al*.^23^ and refer to humans (red), macaques (black), and chimpanzees (blue). Each point represents an individual (25 humans, 31 macaques, and 12 chimpanzees) and lines show lowess fittings^47^. Lighter lines represent the 2.5 and 97.5 confidence intervals calculated using 1000 bootstrap replicates. Age (x-axis) is reported in log_2_ scale and *REST* expression level in log scale.

During human lifespan, *REST* expression increases in the nucleus of aging PFC and hippocampal neurons^8^. To investigate the effect of age on *REST* expression in other primates and compare it with humans, we retrieved data from a study that analyzed mRNA expression in two brain regions (PFC and cerebellar cortex) in humans, macaques, and chimpanzees^23^. In order to assess whether *REST* expression deviates from a null expectation of constant levels throughout lifespan, we applied a bootstrap approach to calculate confidence intervals. Results indicated that in the cerebellar cortex, *REST* expression has a similar pattern in the three species, with a higher expression after birth and a progressive decline with aging (Fig. 2A). Conversely, different expression dynamics were observed in the PFC, with humans showing a decline after birth followed by a plateau in childhood and a steep increase after adolescence. In chimpanzees and macaques *REST* expression tends to increase throughout most of the lifespan (Fig. 2B).

### REST polymorphisms and hippocampal volume loss

We analyzed the possible correlation between *REST* variants and hippocampal volume loss in a group of 99 Italian subjects with AD *continuum*: 60 “clinical AD” and 39 “preclinical AD” subjects according to disease stage^24^. All these subjects underwent MRI evaluation and bilateral hippocampal volumetries were computed from MRI T1-3D high-resolution images as well-established structural imaging markers for AD staging^24^.

Two *REST* variants were genotyped in all patients: rs3796529, located in the VNTR and previously reported as protective for hippocampal atrophy in MCI and AD^10, 11^, and rs2227902, in linkage disequilibrium with the exon 4 VNTR in European populations^12^.

To address their contribution to hippocampal atrophy, linear regression analysis was performed using age (at MRI examination), sex, *APOE* genotype, and volumetric scaling factor calculated by SIENAX as covariates.

We observed no association with hippocampal volume for rs3796529. Conversely, the minor T allele at rs2227902 appeared to be associated with right hippocampus volume loss (Table 3).

**Table 3:** Association of *REST* variants with Left and Right Hippocampus volumes.

Variant	Allele	Left Hippocampus		Right Hippocampus	
		beta^a^	*p value* ^*b*^ (*FDR correction)*	beta^a^	*p value* ^*b*^ (*FDR correction)*
rs3796529	T	0.1188	0.235 (0.235)	−0.0027	0.978 (0.978)
rs2227902	T	−0.1782	0.076 (0.152)	−0.2623	0.008 (0.016)

^a^Regression coefficient; ^b^ *p value* from linear regression.

Haplotype analysis using the same covariates detected a haplotype significantly associated with right hippocampus volume reduction (*p value* = 0.008). The predisposing haplotype includes the rs2227902 T allele and the major allele (C allele) at rs3796529.

## Discussion

Primate genomes have evolved to display many more REST binding sites compared to other mammals, and several recent RE1 elements regulate neuronal genes^13^. Herein we show that, during primate evolution, *REST* evolved under strong positive selection. Indeed, using the integration of multiple methods to limit false positives, we identified 22 sites that were targeted by diversifying selection. Additional selected sites in REST were specific for the human or gorilla lineages. We exclude that the relatively large number of positively selected sites is due to a relaxation of functional constraint rather than to positive selection. In fact, evolutionary fingerprinting identified two major selective classes of negatively and positively selected sites, with no indication of a class of sites close to evolutionary neutrality. Moreover, the distribution of selection coefficients in *Homininae* indicated that the majority of codons are evolving under purifying selection.

None of the selected sites we identified involve the zinc-finger motifs that directly interact with DNA. Conversely, several selected sites are located within the lysine-rich and proline-rich regions of the protein. Additional sites cluster in a C-terminal portion of unspecified function. An interesting possibility is that selected sites modulate REST binding to other co-factors, in turn resulting in a species-specific regulation of gene expression. Recent reports have suggested that REST should be regarded as a platform for the assembly of multiple factors, which differ in a cell type- and genomic context-dependent manner^5, 25, 26^. Different configurations of REST co-factors have been observed^26^ and the REST interactome consists of at least 200 proteins in human cell lines^27^. Unfortunately, the molecular details of these interactions are largely unknown, making it impossible to assess whether selection acted to modulate protein-protein interaction affinities.

In this respect, it is worth drawing a parallel with FOXP2, another transcription factor that was targeted by positive selection in the human lineage and is central for neurodevelopment. Two human-specific substitutions were identified in *FOXP2* ^28, 29^. Although these changes are not located in the DNA binding region, they do confer differential transcriptional regulation *in vitro* ^30^. Because human and chimpanzee FOXP2 proteins bind two co-factors (FOXP1 and FOXP4) with similar affinity^30^, it is unclear whether the two selected sites modulate transcription of downstream targets by differential binding to other co-factors or via distinct mechanisms.

In any case, by analogy with FOXP2, the positively selected sites in REST may well modulate the transcription of downstream targets, although the underlying molecular details remain to be elucidated. This might also explain the dynamic expansion that occurred at the level of RE1 elements during primate evolution, as changes in transactivation activity are expected to expose target sequences to novel evolutionary drives, as again suggested for FOXP2 and its binding sites^15^.

We identified six sites in REST that were targeted by positive selection specifically in the human lineage. These changes arose before the split of the *Homo sapiens* lineage from Neanderthals, indicating that the ensuing phenotypic changes were shared between modern and archaic humans.

Given the peculiarity of human cognition and the observed association of *REST* variants with cognitive abilities (at least in adults)^8^, it is tempting to speculate that the human-specific changes contributed to the evolution of human intelligence. However, some sobering remarks should be mentioned. First, positively selected changes in non-human primates were previously described at genes involved in developmental dyslexia^29^. Clearly, these changes did not provide these primates with reading abilities, indicating that inference of the phenotypic effects of selected sites is challenging. Second, although REST has been extensively studied in relation to neurogenesis and neuroprotection, this transcription factor is ubiquitously expressed and contributes to the regulation of important physiological functions, including vascular smooth muscle cell proliferation^31^, regulation of fetal cardiac gene expression^32^, maintenance of a pluripotent state of embryonic stem cells^5^, and oncogenesis^33^. Third, several works indicated that Herpes Simplex Virus 1 (HSV-1), a neurotropic human virus, has evolved the ability to exploit the CoREST/REST repressor complex to regulate its gene expression^34^. Because viruses related to HSV-1 infect non-human primates^35^, the positive selection signal we identified may result from a conflict between primate hosts and herpes simplex viruses.

Therefore, the ultimate phenotype(s) targeted by natural selection may be related to these diverse functions and not necessarily to neurodevelopment or neuroprotection. As for this latter, the effect of REST has been mainly described in elderly subjects^8^ and natural selection is inefficient or weak for phenotypes with post-reproductive onset.

Although the selective pressures responsible for shaping REST evolution, as well as the phenotypic consequences of such selection remain to be determined, it is clear that this transcription factor plays an important role during human aging.

*REST* levels increase progressively in the nuclei of hippocampal and brain cortical neurons in healthy aging humans. This leads to the upregulation of protective genes and to the downregulation of genes related to neuronal degeneration^8^. In fact, *REST* levels in PFC neurons from elderly subjects positively correlate with cognitive and memory preservation^8^. Interestingly, our analysis of *REST* expression in chimpanzees and macaques indicated that the age-dependent up-regulation of *REST* expression in the PFC, but not in the cerebellum, is specific for humans.

In neurodegenerative diseases, REST has often been observed within cytoplasmatic autophagosomes, and its levels show no increase in the nuclei of hippocampal neurons. As hippocampal atrophy is one of the prominent features of AD, and *REST* expression is reduced in hippocampal neurons of AD patients^8^, it was suggested that *REST* may be associated with the rate of change in hippocampal volume.

Recently, the major allele of the P797L variant (rs3796529) was associated with right hippocampal loss in MCI subjects^10^ and in AD^11^. This variant is located in the *REST* region where several selected sites also map. This association was however questioned by data from the ENIGMA consortium^36^.

Thus, we investigated the role of two *REST* variants (rs3796529 and rs2227902) as modulators of hippocampal volume loss in AD *continuum*. Contrary to the report from Nho and colleagues^10, 11^, we observed no effect of the rs3796529 variant on hippocampal volume loss/preservation; instead, the minor allele of rs2227902 was significantly associated with right hippocampal volume reduction in AD. Nonetheless, a haplotype carrying the minor allele of rs2227902 and the major allele of rs3796529 was found to predispose to hippocampal volume loss, partially in agreement with the results by Nho and coworkers^10, 11^.

However, we note that the size of the AD population we analyzed is small and, consequently, the results reported herein will need independent validation in additional cohorts. Also, the functional effect of the P797L change is presently unknown and no study analyzed the biochemical differences (if any) of REST molecules carrying 4 or 5 copies of the VNTR. Addressing these issues will likely be instrumental to gain insight into the role of *REST* variants in neuropreservation/neurodegeneration.

Overall, these findings, together with previous data, confirm a role of REST in hippocampal atrophy/preservation in neurogenerative disorders. These observation also indicate REST as a promising target for neuroprotective strategies for neurodegenerative disorders, in particular for AD.

## Methods

### Evolutionary analysis in Primates

Primates coding sequences were retrieved from the National Center for Biotechnology Information database (http://www.ncbi.nlm.nih.gov, last accessed October 31, 2015). A list of species and of GenBank accession numbers is available as Supplementary Table S1.

DNA alignments were performed using the RevTrans 2.0 utility^37^. Genetic variability that is generated by recombination can be mistaken as positive selection^38^; thus, to limit false positives, alignments were screened for the presence of recombination breakpoints using Genetic Algorithm Recombination Detection (GARD)^39^. GARD is a program that uses phylogenetic incongruence among segments of a sequence alignment to detect the best-fit number and location of recombination breakpoints^39^. No significant breakpoint (*p value* < 0.01) was detected.

The average non-synonymous substitution/synonymous substitution rate (dN/dS; ω) was estimated using SLAC (Single Likelihood Ancestor Counting)^16^, a tool from the Hyphy package based on a codon substitution matrix and ancestral state reconstruction.

We used the PAML (Phylogenetic Analysis by Maximum Likelihood) software to detected positive selection^19^. The *codeml* NSsite models that allow (M2a, M8) or disallow (M1a, M7) a class of sites to evolve with ω > 1 were fitted to the data using different codon frequencies model: the F3X4 model (codon frequencies estimated from the nucleotide frequencies in the data at each codon site) and the F61 model (frequencies of each of the 61 non-stop codons estimated from the data)^18, 19^. For these analyses, phylogenetic trees were reconstructed using the program phyML with a maximum-likelihood approach, a General Time Reversible (GTR) model plus gamma-distributed rates and 4 substitution rate categories^40^.

Positively selected sites were identified using the following methods: (1) the Bayes Empirical Bayes (BEB) analysis (with a cutoff of 0.90), which calculates the posterior probability that each codon is from the site class of positive selection (under model M8)^41^; (2) the Random Effects Likelihood (REL)^16^, which models variation in nonsynonymous and synonymous rates across sites according to a predefined distribution, with the selection pressure at an individual site inferred using an empirical Bayes approach; (3) Fast Unbiased Bayesian AppRoximation (FUBAR)^42^, an approximate hierarchical Bayesian method that generates an unconstrained distribution of selection parameters to estimate the posterior probability of positive diversifying selection at each site in a given alignment (with a cutoff ≥0.90).

To be conservative, we considered a site under positive selection if it was detected by at least two methods.

To investigate whether episodic positive selection acted on the primate phylogeny, we applied the branch-site unrestricted statistical test for episodic diversification (BUSTED)^16, 20^. BUSTED is designed to detect the action of episodic positive selection that is acting on a subset of branches in the phylogeny at a proportion of sites within the alignment.

GARD, BUSTED, REL, FUBAR and SLAC, as well as evolutionary fingerprinting^21^ analyses were performed either through the DataMonkey server^43^ (http://www.datamonkey.org) or run locally (through the HyPhy suite)^44^.

### Population genetics-phylogenetics analysis

For the population genetics-phylogenetics analysis, genotype data from the Phase 1 of the 1000 Genomes Project were retrieved from the dedicated website (http://www.1000genomes.org/)^45^; in particular, SNP information were retrieved for individuals of three human populations: African (Yoruba), European, and East Asian (Chinese). Ancestral sequences were reconstructed by parsimony from the human, chimpanzee, orangutan, and macaque sequences.

For the chimpanzee and gorilla analyses, we used SNP information from 25 and 27 individuals, respectively^46^.

Analyses were performed with gammaMap^22^, that uses intra-specific variation and inter-specific diversity to estimate the distribution of population-scaled selection coefficients (γ) along coding regions. gammaMap classifies γ values into 12 categories, ranging from strongly beneficial (γ = 100) to inviable (γ = −500), with γ equal to 0 indicating neutrality. In the analysis, we assumed θ (neutral mutation rate per site), k (transitions/transversions ratio), and T (branch length) to vary among genes following log-normal distributions. For p (the probability that adjacent codons share the same population-scaled selection coefficient) we assumed a uniform distribution. We set the neutral frequencies of non-STOP codons to 1/61. For population-scaled selection coefficients we considered a uniform Dirichlet distribution with the same prior weight for each selection class. For each gene, two Markov Chain Monte Carlo runs of 100,000 iterations each were run with a thinning interval of 10 iterations. Runs were compared to assess convergence and merged to obtain posterior probabilities. To be conservative, we declared a codon to be targeted by positive selection when the cumulative posterior probability of γ ≥ 1 was >0.80.

### VNTR analysis

A VNTR (Variable Number Tandem Repeat) with four or five copies has been described in the exon 4 of human *REST* gene^12^. In order to verify if the VNTR is polymorphic in chimpanzee, the genomic DNA of twelve *Pan troglodytes* was used as a template for *REST* exon 4 amplification and sequencing. We also analyzed VNTR status in one *Gorilla gorilla*. In particular, the DNAs, kindly provided by the Gene Bank of Primates (Primate Genetics, Germany), were amplified by PCR (primer Forward:CTGCTCAGATGGACCCTCCT; primer Reverse:GTGCCCTTTCACTCTGCATGT), and then treated with ExoSAP-IT (USB Corporation Cleveland Ohio, USA). Purified PCR products were directly sequenced on both strands with a Big Dye Terminator sequencing Kit (v3.1, Thermo Fisher Scientific), and run on an Applied Biosystems ABI 3130 XL Genetic Analyzer (Thermo Fisher Scientific).

### REST expression in primates

*REST* mRNA expression levels in the prefrontal and cerebellar cortices were retrieved from a large-scale study that analyzed humans, macaques, and chimpanzees^23^.

To evaluate whether *REST* expression in the three primate species is different during lifespan, we applied a resampling approach. Specifically, for each species, we performed 1000 random reshuffles of expression values; this corresponds to a null hypothesis of constant *REST* expression during lifespan. For each reshuffle, a lowess fitting^47^ was calculated and confidence intervals were retrieved form the 2.5% and 97.5% percentiles of the resulting distributions.

### Patients, genotyping, MRI data acquisition, and statistical analysis

A total of 99 Italian subjects with AD *continuum* were consecutively recruited at the Neurology Department of IRCCS Don C. Gnocchi Foundation in Milano, Italy.

The study was in accordance with the principles of the 1975 Declaration of Helsinki and was approved by the Institutional Review Boards at the Don C. Gnocchi Foundation ONLUS.

All patients gave written informed consent.

Subjects were clinically classified and distinguished according to stage^24^ in: 60 “clinical AD” (23 males, 37 females) and 39 “preclinical AD” (19 males, 20 females). The mean age of “clinical AD” patients was 76.41 years (5.53 standard deviation; age range 63–89 years) and of “preclinical AD” subjects was 74.10 (6.06 standard deviation; age range 62–85). All subjects underwent complete medical and neurological evaluation, laboratory analysis, MRI scan, and other investigations – when necessary (e.g. EEG, SPET scan, CSF examination, etc.) – to exclude reversible causes of dementia.

In all subjects, we analysed two variants in *REST*: rs3796529, previously associated with hippocampal volume loss^10, 11^, and rs2227902. This latter is in linkage disequilibrium with VNTR in exon 4 in the European ancestry population and has been associated with general cognitive ability^12^.

The two *REST* variants were genotyped by allelic discrimination real-time PCR, using predesigned TaqMan probe assays (ThermoFisher Scientific). Reactions were performed using TaqMan Genotyping Master Mix in an ABI 9700 analyzer (Applied Biosystems). Genotyping rate was >0.97 for both variants.

All subjects underwent also MRI examination (1.5 T Siemens AVANTO). Hippocampal volume data have been extracted for each subject from high-resolution T1 3D images (MPRAGE; TR/TE = 1900/3.37 ms, FoV = 192 mm × 256 mm, in-plane resolution 1 mm × 1 mm, slice thickness = 1 mm, number of axial slices = 176) using FSL-FIRST segmentation method^48^. Intracranial brain volume data were obtained using SIENAX^49^, part of FSL^50^. In particular, SIENAX was used to estimate brain tissue volume normalised for subject skull size.

Genetic association with hippocampal volumes was performed separately for right and left hippocampal volumes. In particular, we applied linear regression analysis using genotypes or haplotypes as the independent predictor variables with age (at MRI examination), sex, *APOE* genotype, and volumetric scaling factor calculated by SIENAX as covariates. False Discovery Rate (FDR) correction was applied to account for the two tested variants. All analyses were performed using PLINK^51^.

## Electronic supplementary material

Supplementary Material
