# Supplementary material for: REST, a master regulator of neurogenesis, evolved under strong positive selection in humans and in non human primates
Source: Sci Rep. 2017 Aug 25;7:9530. doi: 10.1038/s41598-017-10245-w (PMC5573535; doi:10.1038/s41598-017-10245-w)
Supplement: Supplementary file 1 — Supplementary Material [file 41598_2017_10245_MOESM1_ESM.pdf]

Supplementary Information

REST, a master regulator of neurogenesis, evolved under strong positive selection in humans and in non human primates

Alessandra Mozzi, Franca Rosa Guerini, Diego Forni, Andrea Saul Costa, Raffaello Nemni, Francesca Baglio, Monia Cabinio, Stefania Riva, Chiara Pontremoli, Mario Clerici, Manuela Sironi, Rachele Cagliani

Supplementary Table 1. List of primate species

**Common Name**	***Scientific Name***	**NCBI accession number**
Nancy Ma's night monkey	*Aotus nancymaae*	XM_012441134
Marmoset	*Callithrix jacchus*	XM_008993269
Sooty mangabey	*Cercocebus atys*	XM_012036586
Green monkey	*Chlorocebus sabaus*	XM_007998606
Sclater's Angola colobus	*Colobus angolensis palliatus*	XM_011937177
Gorilla	*Gorilla gorilla gorilla*	XM_004038718
Human	*Homo sapiens*	NM_005612
Crab-eating macaque	*Macaca fascicularis*	XM_005555211
Rhesus macaque	*Macaca mulatta*	XM_002804106
Southern pig-tailed macaque	*Macaca nemestrina*	XM_011710747
Mandrill	*Mandrillus leucophaeus*	XM_011998457
Mouse lemur	*Microcebus murinus*	XM_012758275
Gibbon	*Nomascus leucogenys*	XM_003268406
Bushbaby	*Otolemur garnettii*	XM_012810363
Bonobo	*Pan paniscus*	XM_003806496
Chimpanzee	*Pan troglodytes*	XM_003310370
Olive-baboon	*Papio anubis*	XM_009207025
Orangutan	*Pongo abelii*	XM_002814760
Coquerel's sifaka	*Propithecus coquereli*	XM_012641111
Snub-nosed monkey	*Rhinopithecus roxellana*	XM_010389436
Squirrel monkey	*Saimiri boliviensis*	XM_010344121
